# Role of Vaspin in Human Eating Behaviour

**DOI:** 10.1371/journal.pone.0054140

**Published:** 2013-01-14

**Authors:** Jana Breitfeld, Anke Tönjes, Marie-Therese Gast, Dorit Schleinitz, Matthias Blüher, Michael Stumvoll, Peter Kovacs, Yvonne Böttcher

**Affiliations:** 1 Department of Medicine, University of Leipzig, Leipzig, Germany; 2 IFB Adiposity Diseases, University of Leipzig, Leipzig, Germany; Charité University Medicine Berlin, Germany

## Abstract

**Objective:**

The adipokine vaspin (visceral adipose tissue derived serine protease inhibitor, serpinA12) follows a meal-related diurnal variation in humans and intracerebroventricular vaspin administration leads to acutely reduced food intake in *db/db* mice. We therefore hypothesized that vaspin may play a role in human eating behaviour.

**Materials and Methods:**

We measured serum vaspin concentrations in 548 subjects from a self-contained population of Sorbs (Germany) who underwent detailed metabolic testing including eating behaviour assessments using the three-factor eating questionnaire. In addition, genetic variation within *vaspin* was assessed by genotyping 28 single nucleotide polymorphisms (SNPs) in all study subjects.

**Results:**

Serum vaspin concentrations correlated positively with restraint, disinhibition and hunger (all *P*<0.05), although the correlations did not withstand further adjustments for age, gender and BMI (all *P*>0.05). Independent of observed correlations, genetic variants in *vaspin* were associated with serum vaspin levels but showed no significant association with any of the eating behaviour phenotypes after accounting for multiple testing (*P*≥0.05 after adjusting for age, gender and BMI).

**Conclusion:**

Our data suggest that serum vaspin concentrations might modulate human eating behaviour, which does not seem to be affected by common genetic variation in vaspin.

## Introduction

The visceral adipose tissue derived serine protease inhibitor (vaspin, serpinA12) is an adipokine potentially linking obesity, type 2 diabetes (T2D) and insulin resistance (IR). Vaspin was isolated from visceral adipose tissue of the Otsuka Long-Evans Tokushima fatty (OLETF) rat, an experimental model for T2D [1] and was postulated to have insulin sensitizing effects in state of obesity [2]. Although adipose tissue and liver have been favoured as main target organs of vaspin action [2], [3], other organs can not be excluded either. For instance, along with original studies by Hida *et al.* (2005) showing presence of vaspin in visceral adipose tissue [2], we detected vaspin in the hypothalamus of *db/db* and *C57BL/6* mice as well as in the cerebrospinal fluid and in the stomach of healthy individuals [4]. Moreover, intracerebroventricular vaspin injection resulted in acutely reduced food intake and in sustained improvement of glucose concentrations in *db/db* mice. In line with this, circulating vaspin levels followed a meal-related diurnal variation in humans, similar to that seen for ghrelin [5] and further supporting the so far unrecognized role of vaspin in the regulation of food intake. Interestingly, variation in vaspin concentrations has a strong genetic component as implicated by recent associations between serum vaspin and genetic variants in the *vaspin* locus [6]–[8].

Here, we hypothesized that serum vaspin might be involved in the regulation of human eating behaviour. We therefore tested whether serum vaspin concentrations correlate with human eating behaviour factors assessed by the widely used Three-Factor Eating Questionnaire (TFEQ) [9]. To rule out any unknown confounders in a potential relationship between eating behaviour and circulating vaspin, we further analyzed the associations between human eating behaviour factors and *vaspin* genetic variants.

## Materials and Methods

### Study Subjects and the Three-Factor Eating Questionnaire

A population of the Sorbs from Eastern Germany had been extensively phenotyped for a wide range of metabolic traits as described elsewhere [10]–[12]. In total, 618 Sorbian individuals completed the German version of the TFEQ [9] measuring three different eating behaviour factors affecting food intake: restraint, disinhibition and hunger. Restraint defines the extent of individual cognitive control to limit food intake in order to control body weight (21 items) whereas disinhibition measures the loss of cognitive control resulting in overeating (16 items). Hunger represents perception of hunger or the need of food (14 items). To avoid any confounding by T2D or its treatment, subjects with T2D were excluded from the study (definition of T2D according to ADA criteria [13]). The self-reported frequency of depression in the study population is 10.8% and 7.8% of the subjects were on antidepressant medication. Only one subject (0.2%) suffered from schizophrenia but this subject was on medication. Finally, the study included 548 subjects without T2D who gave written informed consent and whose main characteristics are presented in Table 1. The study has been approved by the ethics committee of the University of Leipzig.

**Table 1:** Main characteristics of the study subjects (N = 548).

	female	male	total
N	346	202	548
age (years)	45±15	47±16	46±16
BMI (kg/m^2^)	25.7±4.9	26.7±3.3	26.1±4.4
serum vaspin (ng/ml)	0.78±3.03	0.33±2.57	0.57±3.09
restraint score	8.9±5.1	6.2±4.0	7.9±4.9
disinhibition score	4.7±3.2	3.7±2.5	4.3±3.0
hunger score	4.2±2.9	3.5±2.6	3.9±2.8
lipid-lowering drugs	7.8%	9.4%	8.4%
beta blockers	11.6%	13.4%	12.2%
ACE inhibitors	4.6%	9.9%	6.6%
angiotensin 2 receptor inhibitors	5.5%	5.4%	5.5%
calcium channel blockers	2.6%	3.5%	2.9%
antithyroid drugs	1.2%	0.5%	0.7%
levothyroxine	14.2%	2.0%	9.7%

Means±standard deviation are given.

### Measurement of Serum Vaspin

Blood samples were taken after an overnight fast and serum was separated by centrifugation. Using the enzyme-linked immunosorbent assay kit by AdipoGen (Seoul, Korea) serum vaspin concentrations were determined according to the manufactureŕs protocol [14]. The assay has a sensitivity of 12 pg/ml and the intra- and inter- assay coefficients of variance were 1.3% to 3.8% and 3.3% to 9.1%, respectively.

### Genotyping of Vaspin Genetic Variants

28 representative single nucleotide polymorphisms (SNPs) have been previously genotyped to completely cover the common genetic variation within *vaspin* [8]. Briefly, the KASPar Assay system was used according to the manufactureŕs protocol (KBioscience Ltd, Hoddesdon, United Kingdom) and detection was done using the ABI PRISM 7500 Sequence Detecting System. To assess genotyping reproducibility, a random ∼5% selection of samples were re-genotyped for all SNPs; all genotypes matched initial designated genotypes.

### Statistical Analysis

Statistical analysis was done using the PASW statistics version 20.0.1 (SPSS, Inc.; Chicago, IL). All non-normally distributed variables were logarithmically transformed to approximate normal distribution. Linear regression analysis was used to analyse the relationship of serum vaspin and eating behaviour and to assess a potential association between genetic variants and eating behaviour. Gender-genotype interaction as well as gender-vaspin-interaction was analyzed via linear regression analysis. The presented *P*-values were calculated in additive mode of inheritance and were adjusted for age, gender and body-mass-index (BMI). *P*-values <0.05 but not reaching statistical significance levels according to Bonferroni correction for multiple testing (required *P*<0.002 considering the 28 SNPs) were considered to be of nominal statistical significance. *P*-values are provided without the Bonferroni correction and effect directions are standardized to the positive strand and the minor allele.

Power calculation was done using the Quanto 1.2.4 computer program [15].

## Results

### Correlation of Serum Vaspin and Eating Behaviour

**Figure 1:**
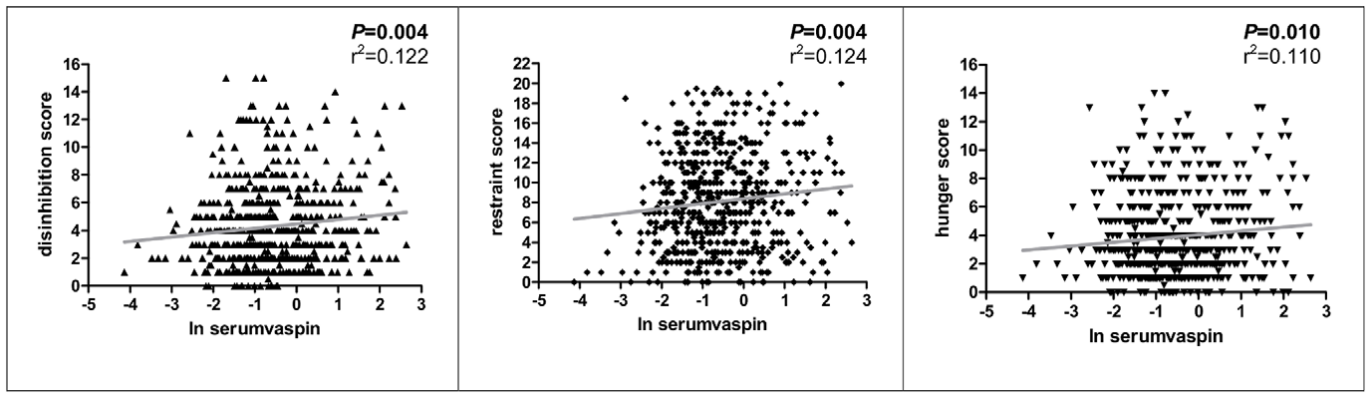
Correlations of eating behaviour phenotypes with serum vaspin concentrations (N = 548). *P*-values were calculated using linear regression.

Serum vaspin concentrations positively correlated with restraint (*P* = 0.004), disinhibition (*P* = 0.004) and hunger (*P* = 0.010) (Figure 1; Table 2). After adjusting for age and BMI the correlation with restraint (*P* = 4.3×10^−4^) and disinhibition (*P* = 0.013) remained significant, but this did not withstand further adjustment for gender (Table 2). It is of note that medication did not affect the initially observed relationship between eating behaviour and circulating vaspin in any of the tested multivariate linear models (Table 2). Moreover, the data remained materially unchanged even after excluding subjects on medication from the analyses (data not shown).

**Table 2:** Multivariate linear regression analyses of eating behaviour phenotypes with anthropometric parameters in the Sorbs (N = 548).

	restraint		disinhibition		hunger	
	*P*-value	ß	*P*-value	ß	*P*-value	ß
**model 1**						
serum vaspin	**0.004**	0.536	**0.004**	0.325	**0.010**	0.276
**model 2**						
serum vaspin	**0.001**	0.629	**0.045**	0.224	0.082	0.183
age	0.001	0.044	3.7×10^−9^	−0.048	8.3×10^−9^	−0.044
**model 3**						
serum vaspin	**0.001**	0.608	**0.001**	0.379	**0.009**	0.284
BMI	1.5×10^−4^	4.897	7.2×10^−6^	3.584	0.678	0.314
**model 4**						
serum vaspin	**0.004**	0.525	**0.003**	0.336	**0.008**	0.284
medication	0.071	0.797	0.008	−0.72	0.032	−0.552
**model 5**						
serum vaspin	0.524	0.122	0.103	0.199	0.085	0.198
gender	6.8×10^−9^	−2.639	0.005	−0.808	0.063	−0.5
**model 6**						
serum vaspin	**4.3**×**10** ^−**4**^	0.651	**0.013**	0.263	**0.055**	0.2
age	0.049	0.029	4.9×10^−19^	−0.078	5.8×10^−11^	−0.056
BMI	0.008	3.73	8.2×10^−16^	6.725	0.001	2.557
**model 7**						
serum vaspin	0.264	0.215	0.406	0.099	0.345	0.106
age	0.001	0.045	3.1×10^−9^	−0.048	8.2×10^−9^	−0.044
gender	4.4×10^−9^	−2.646	0.004	−0.801	0.06	−0.491
**model 8**						
serum vaspin	0.359	0.173	**0.053**	0.232	0.078	0.203
BMI	4.5×10^−6^	5.777	1.1×10^−6^	3.882	0.529	0.477
gender	2.8×10^−10^	−2.86	0.001	−0.968	0.051	−0.53
**model 9**						
serum vaspin	0.258	0.216	0.403	0.093	0.347	0.105
age	0.087	0.024	2.8×10^−20^	−0.08	2.4×10^−11^	−0.057
gender	4.8×10^−10^	−2.82	2.9×10^−5^	−1.097	0.018	−0.619
BMI	0.001	4.786	9.8×10^−18^	7.136	0.001	2.788
**model 10**						
serum vaspin	**0.001**	0.647	**0.009**	0.279	0.056	0.202
BMI	0.009	3.732	4.5×10^−16^	6.856	0.002	2.56
age	0.088	0.027	3.7×10^−15^	−0.074	1.7×10^−9^	−0.055
medication	0.914	0.053	0.223	−0.344	0.939	−0.022
**model 11**						
serum vaspin	0.260	0.215	0.338	0.107	0.332	0.109
age	0.083	0.026	1.1×10^−15^	−0.074	1.4×10^−9^	−0.055
gender	2.1×10^−10^	−2.905	1.3×10^−5^	−1.156	0.018	−0.627
BMI	3.6×10^−4^	4.981	2.5×10^−18^	7.352	0.001	2.829
medication	0.481	−0.338	0.076	−0.499	0.708	−0.105

Data represent subjects without type 2 diabetes. *P*<0.05 (in bold) are presented without correction for multiple testing. BMI = body-mass index.

### Association of Vaspin Genetic Variants and Eating Behaviour

To assess potential association of *vaspin* genetic variants with eating behaviour factors we applied linear regression models adjusted for age, gender and BMI. We observed nominal associations between disinhibition and two *vaspin* SNPs, rs3736806 (*P* = 0.025) and rs4905233 (*P* = 0.046; Table 3). Furthermore, rs8006968, rs17090987 and rs7152269 were nominally associated with hunger (*P* = 0.016, *P* = 0.038 and *P* = 0.053; Table 3). However, none of these associations withstood Bonferroni corrections for multiple testing (all *P*>0.002).

**Table 3:** Association of *vaspin* genetic variants with eating behaviour phenotypes (N = 548).

	restraint		disinhibition		hunger	
SNP	*P*-value	ß	*P*-value	ß	*P*-value	ß
rs17094919 C/T	0.546	−0.231	0.067	0.060	0.958	−0.011
rs17752900 C/T	0.936	−0.024	0.200	−0.035	0.219	0.210
rs76624128 T/C	0.742	−0.176	0.418	0.039	0.934	−0.026
rs74077748 G/T	0.253	−0.853	0.062	0.116	0.742	−0.129
rs8006968 T/A	0.760	−0.109	0.852	0.006	**0.016**	−0.496
rs17090987 C/G	0.829	−0.095	0.877	−0.006	**0.038**	−0.529
rs1951017 T/C	0.666	0.191	0.495	0.028	0.670	0.110
rs17752833 C/T	0.905	−0.050	0.708	0.014	0.950	0.015
rs7158068 T/C	0.256	−0.344	0.246	0.032	0.296	−0.182
rs10145558 A/G	0.562	−0.314	0.373	0.044	0.137	−2.011
rs1951007 T/C	0.970	0.012	0.652	0.013	0.148	−0.260
rs1956709 A/G	0.329	−0.322	0.289	0.204	0.835	−0.040
rs8015166 T/C	0.331	−0.404	0.943	0.003	0.286	−0.254
rs10146894 A/G	0.603	−0.329	0.609	0.029	0.171	0.497
rs12433651 A/G	0.676	0.178	0.287	0.040	0.589	0.128
rs11625995 A/T	0.901	−0.036	0.232	0.031	0.881	−0.025
rs11625941 T/A	0.649	0.175	0.933	0.003	0.162	0.310
rs4905211 G/A	0.256	−0.341	0.204	0.035	0.695	−0.069
rs1012808 A/G	0.685	0.156	0.966	0.009	0.536	−0.138
rs2236240 G/A	0.869	−0.088	0.205	0.062	0.662	−0.136
rs7152296 G/A	0.926	−0.039	0.697	−0.015	**0.053**	−0.464
rs2236241 T/C	0.675	0.167	0.528	0.023	0.077	0.403
rs2236242 A/T	0.590	0.153	0.454	−0.019	0.189	0.217
rs1998207 C/A	0.813	−0.083	0.225	0.038	0.202	0.257
rs3736806 A/G	0.122	−0.940	**0.025**	0.123	0.937	−0.028
rs4900233 G/A	0.236	−0.750	**0.046**	0.113	0.601	−0.190
rs3736803 G/A	0.694	−0.172	0.348	−0.037	0.059	−0.460
rs11626701 G/A	0.940	−0.024	0.345	0.027	0.276	−0.198

Data represent subjects without type 2 diabetes. *P*-values were calculated by linear regression analysis, after adjusting for age, gender and BMI in the additive mode of inheritance and are presented without correction for multiple testing. *P*-values <0.05 are in bold. ß (regression coefficient) is standardized to the minor allele. SNP = single nucleotide polymorphism; BMI = body mass index.

We further analyzed the gender-SNP interaction for all 3 factors, which reached statistical significance with *P*-values up to 2.3×10^−7^. Similar to the analyses in the entire cohort, gender stratified analyses did not reveal any significant association between the SNPs and eating behaviour phenotypes (Table S1).

## Discussion

In the present study we hypothesized that vaspin might play a role in the regulation of human eating behaviour. We found significant correlations between circulating vaspin concentrations and eating behaviour factors assessed by the German version of the Three-Factor Eating Questionnaire [9]. However genetic variants within the *vaspin* gene which associate with vaspin concentrations [8] did not show significant association with eating behaviour.

Vaspin was originally identified as an adipokine potentially linking obesity, insulin resistance and T2D [2]. Based on its strong homology with alpha_1_-antitrypsin it belongs to broadly distributed serpins, a protein superfamily of serine protease inhibitors of ∼500 genes, and is identical to serpinA12. So far, adipose tissue has been favoured as a target organ of vaspin action, as vaspin was isolated from visceral white adipose tissue of the OLETF rat and vaspin administration to obese mice led to the reversal of altered expression of genes relevant to insulin resistance [2]. However, very recent data by Nakatsuka *et al.* suggest that vaspin ameliorates ER stress in liver as a ligand for cell surface GRP78/MTJ-1 complex [3]. To further expand the list of organs relevant to vaspin’s action, our present study shows a relationship between vaspin and human eating behaviour. Even though this relationship is mere association and does not allow any conclusion with regards to causality, our data are in line with recent findings in mice, where intracerebroventricular vaspin administration resulted in acute reduction of food intake and sustained amelioration of plasma glucose [4]. Furthermore, vaspin injection into the arcuate nucleus of the hypothalamus in rats decreased food intake 24 h after vaspin administration, which seemed to be mediated by decreased gene expression of the orexigenic neuropeptide Y (NPY) and increased expression of the anorexigenic POMC (proopiomelanocortin), both involved in the central modulation of feeding [16]. It is of note that vaspin has been detected in the human cerebrospinal fluid as well and the mRNA expression of *vaspin* has been shown in the stomach of obese human subjects after gastric sleeve resection [4]. Altogether, correlations of vaspin with eating behaviour (hunger, disinhibition and restraint) suggest a previously unrecognized role of vaspin in the cognitive control of food intake. It needs to be mentioned that the three eating behaviour factors are not entirely independent from each other. The positive correlations between eating behaviour phenotypes and serum vaspin concentrations indicate that vaspin might potentiate hunger feeling, which would consequently lead to an increased food intake. Under normal physiologic conditions the increased food intake would be under higher cognitive control mirrored by a higher restraint score. However, losing this control would lead to a higher disinhibition and would consequently further promote increased food intake.

Our findings deserve particular attention in the context of data by Jeong *et al.* who reported that serum vaspin concentrations raised up prior to an anticipated meal even if the study subjects remained fasted [5]. Altogether these studies support the potential neuronal function of vaspin and despite missing direct evidence one might hypothesize that vaspin degrades an anti-orexigenic factor.

It is of note however that the correlations can not be supported by the associations of genetic variants in *vaspin* with assessed factors of eating behaviour although we could recently show that *vaspin* polymorphisms were associated with circulating vaspin concentrations as well [8]. We are aware that due to the small sample size we had only limited statistical power to detect any SNP effects. Given minor allele frequencies ranging from 5–50%, we had 80% power (alpha = 0.05) to detect genotypic differences in restraint scores ranging from 11–25%, in disinhibition scores from 12–27% and hunger scores from 12–28%. Therefore, due to the lack of power, smaller effects could have been missed easily in our study. Although replication of our findings in independent cohorts would be desirable, identification of the causal variant(s) controlling circulating vaspin levels will still be inevitable to establish a causative link between serum vaspin, eating behaviour and genetic variation.

In conclusion, we show that circulating vaspin correlates with human eating behaviour, which seems to be unaffected by genetic variation within the *vaspin* gene. Our data suggest that the previously reported association between circulating vaspin and metabolic traits might be mediated by vaspin effects on human eating behaviour.

## Supporting Information

Table S1 Gender-stratified *vaspin*-SNP associations with eating behaviour phenotypes (N = 548).
(DOC)
