# Supplementary material for: Role of Vaspin in Human Eating Behaviour
Source: PLoS One. 2013 Jan 14;8(1):e54140. doi: 10.1371/journal.pone.0054140 (PMC3544656; doi:10.1371/journal.pone.0054140)
Supplement: Table S1 — Gender-stratified vaspin-SNP associations with eating behaviour phenotypes (N = 548). (DOC) [file pone.0054140.s001.doc]

**Table S1:** Gender-stratified *vaspin*-SNP associations with eating behaviour phenotypes (N=548).

|  | **restraint** | | | | **disinhibition** | | | | **hunger** | | | |
| --- | --- | --- | --- | --- | --- | --- | --- | --- | --- | --- | --- | --- |
| **SNP** | ***P female*** | **ß** | ***P male*** | **ß** | ***P female*** | **ß** | ***P male*** | **ß** | ***P female*** | **ß** | ***P male*** | **ß** |
| rs17094919 C/T | 0.982 | 0.011 | 0.277 | 0.617 | 0.684 | -0.108 | **0.007** | -0.834 | 0.399 | -0.235 | 0.210 | 0.403 |
| rs17752900 C/T | 0.894 | 0.056 | 0.693 | -0.165 | 0.342 | 0.218 | 0.197 | -0.302 | 0.143 | 0.334 | 0.888 | 0.036 |
| rs76624128 T/C | 0.992 | -0.007 | 0.481 | 0.586 | 0.973 | -0.014 | 0.167 | -0.662 | 0.850 | -0.074 | 0.977 | 0.015 |
| rs74077748 G/T | 0.685 | -0.390 | **0.036** | -2.491 | 0.785 | 0.128 | 0.085 | 1.141 | 0.576 | -0.278 | 0.811 | 0.160 |
| rs8006968 T/A | 0.810 | -0.115 | 0.756 | -0.162 | 0.203 | -0.352 | 0.115 | 0.466 | **0.023** | -0.607 | 0.374 | -0.279 |
| rs17090987 C/G | 0.665 | 0.251 | 0.823 | -0.145 | 0.207 | 0.421 | 0.190 | -0.492 | 0.071 | -0.593 | 0.324 | -0.388 |
| rs1951017 T/C | 0.744 | 0.195 | 0.699 | 0.244 | 0.782 | 0.096 | 0.194 | 0.477 | 0.928 | -0.031 | 0.481 | 0.271 |
| rs17752833 C/T | 0.865 | -0.101 | 0.966 | 0.024 | 0.891 | -0.046 | 0.368 | -0.290 | 0.698 | -0.128 | 0.587 | 0.188 |
| rs7158068 T/C | 0.319 | 0.415 | 0.531 | 0.268 | 0.533 | 0.149 | 0.322 | -0.238 | 0.285 | -0.249 | 0.711 | -0.096 |
| rs10145558 A/G | 0.744 | 0.239 | 0.585 | 0.416 | 0.816 | 0.099 | 0.315 | -0.443 | 0.883 | -0.061 | 0.673 | -0.195 |
| rs1951007 T/C | 0.898 | -0.055 | 0.995 | 0.003 | 0.716 | -0.088 | 0.817 | 0.061 | 0.354 | -0.217 | 0.284 | 0.296 |
| rs1956709 A/G | 0.472 | -0.314 | 0.482 | -0.034 | 0.864 | -0.043 | **0.034** | 0.598 | 0.981 | -0.006 | 0.576 | -0.166 |
| rs8015166 T/C | 0.350 | 0.528 | 0.695 | 0.231 | 0.341 | 0.307 | 0.110 | -0.527 | 0.347 | -0.297 | 0.614 | -0.179 |
| rs10146894 A/G | 0.730 | 0.301 | 0.693 | 0.347 | 0.981 | -0.012 | 0.458 | -0.367 | 0.342 | 0.462 | 0.393 | 0.454 |
| rs12433651 A/G | 0.981 | 0.014 | 0.531 | 0.371 | 0.817 | 0.073 | 0.192 | -0.429 | 0.573 | 0.176 | 0.762 | 0.107 |
| rs11625995 A/T | 0.789 | 0.105 | 0.914 | -0.044 | 0.924 | 0.022 | **0.037** | -0.493 | 0.639 | -0.104 | 0.742 | 0.082 |
| rs11625941 T/A | 0.847 | -0.097 | 0.159 | 0.804 | 0.826 | -0.064 | 0.253 | 0.373 | 0.350 | 0.266 | 0.285 | 0.369 |
| rs4905211 G/A | 0.358 | -0.367 | 0.356 | -0.408 | 0.870 | 0.038 | 0.513 | 0.167 | 0.912 | -0.025 | 0.631 | -0.129 |
| rs1012808 A/G | 0.995 | 0.004 | 0.454 | 0.411 | 0.379 | -0.262 | 0.159 | 0.442 | 0.541 | -0.179 | 0.794 | -0.087 |
| rs2236240 G/A | 0.486 | 0.514 | 0.175 | -0.979 | 0.810 | 0.103 | 0.390 | 0.368 | 0.576 | -0.234 | 0.870 | -0.074 |
| rs7152296 G/A | 0.593 | -0.308 | 0.525 | 0.369 | 0.089 | -0.559 | 0.238 | 0.384 | **0.051** | -0.624 | 0.558 | -0.205 |
| rs2236241 T/C | 0.395 | 0.454 | 0.567 | -0.329 | 0.663 | 0.133 | **0.051** | 0.618 | 0.256 | 0.338 | 0.164 | 0.479 |
| rs2236242 A/T | 0.849 | 0.073 | 0.111 | -0.660 | 0.322 | -0.217 | 0.249 | 0.276 | 0.113 | 0.342 | 0.954 | -0.014 |
| rs1998207 C/A | 0.709 | 0.173 | 0.261 | -0.575 | 0.431 | 0.209 | 0.073 | 0.525 | 0.297 | 0.272 | 0.481 | 0.214 |
| rs3736806 A/G | 0.310 | 0.839 | 0.171 | 1.163 | 0.071 | -0.863 | 0.849 | -0.094 | 0.700 | 0.181 | 0.517 | -0.335 |
| rs4900233 G/A | 0.581 | -0.487 | 0.170 | -1.180 | 0.232 | 0.602 | 0.629 | 0.233 | 0.795 | -0.128 | 0.643 | -0.238 |
| rs3736803 G/A | 0.824 | -0.135 | 0.238 | 0.714 | 0.172 | 0.452 | 0.055 | 0.643 | **0.038** | -0.674 | 0.823 | -0.081 |
| rs11626701 G/A | 0.950 | -0.027 | 0.900 | -0.054 | 0.500 | -0.170 | **0.021** | 0.556 | 0.209 | -0.309 | 0.943 | -0.019 |

Data represent subjects without type 2 diabetes. *P*-values were calculated by linear regression analysis, after adjusting for age, gender and BMI in the additive mode of inheritance and are presented without correction for multiple testing *P*-values <0.05 are in bold. SNP=single nucleotide polymorphism; ß (regression coefficient) is standardized to the minor allele; BMI=body mass index.
